# Supplementary material for: Practitioners’ perspectives on acupuncture treatment for postpartum depression: A qualitative study
Source: PLoS One. 2023 Mar 3;18(3):e0282661. doi: 10.1371/journal.pone.0282661 (PMC9983845; doi:10.1371/journal.pone.0282661)
Supplement: S1 Table — (DOCX) [file pone.0282661.s001.docx]

**Supporting Table 1 Consolidated criteria for reporting qualitative research (COREQ): a 32-item checklist for interviews and focus groups**

| **No. Item** | **Guide questions/description** |
| --- | --- |
| **Domain 1: Research team and reflexivity**  **Personal Characteristics** | |
| 1. Interviewer / facilitator | Which author(s) conducted the interview of focus group?  The first author, based in Shenzhen city, conducted all the interviews. Page 6 |
| 1. Credentials | What were the researcher’s credential? E.g. PhD. MD  The interviewer is a Ph.D. in Chinese medicine. Page 6 |
| 1. Occupation | What was their occupation at the time of the study?  He was a postdoc at the time of the study. Page 6 |
| 1. Gender | Was the researcher male or female?  The interviewer is male. Page 6 |
| 1. Experience and training | What experience or training did the researcher have?  He has had training and experience in qualitative research since 2019. Page 6 |
| **Relationship with participants** | |
| 1. Relationship established | Was a relationship established prior to study commencement?  The interviewer knew10 participants before, 4 participants first met in the interview. Page 7 |
| 1. Participant knowledge of the interviewer | What did the participants know about the researcher? E.g. personal goal, reasons for doing the research  The interviewees were informed about the researchers’ background, occupation and credentials. They were described the purpose of the research, signed the informed consent, and could withdraw from the interviews at any time during the research process. Page 8-9 |
| 1. Interviewer characteristics | What characteristics were reported about the interviewer/facilitator? E.g. Bias, assumptions, reasons and interests in the research topic  The research topic is acupuncture for PPD, from the perspectives of acupuncture practitioners to assess the status quo of acupuncture for PPD and further examine the possible role of acupuncture as a supplementary therapeutic option for PPD. We also aimed to understand the potential barriers and facilitators to the implementation of acupuncture. Page 4 |
| **Domain 2: study design**  **Theoretical framework** | |
| 1. Methodological orientation and theory | What methodological orientation was stated to underpin the study? E.g. grounded theory, discourse analysis, ethnography, phenomenology, content analysis  The study was performed from a constructivist point of view using a descriptive qualitative approach based on the general tenets of naturalistic inquiry. The transcripts were analysed using content qualitative analysis. Page 5 |
| **Participant selection** | |
| 1. Sampling | How were participants selected? E.g. purposive, convenience, consecutive, snowball  The sampling methods of the survey was purposive sampling strategy. Page 6 |
| 1. Method of approach | How were participants approached? E.g. face-to-face, telephone, mail, email  Face-to-face or by telephone depending on the locations of the participants. Page 6 |
| 1. Sample size | How many participants were in the study?  There were 14 participants. Page 6 |
| 1. Non-participation | How many people refused to participate or dropped out? Reasons?  16 people were contacted, One acupuncturist declined, 1 did not respond, and 14 agreed to participate. Page 6 |
| **Setting** | |
| 1. Setting of data collection | Where was the data collected? E.g. home, clinic, workplace  Face-to-face interviews were held in each doctor's office. Page 7 |
| 1. Presence of non-participants | Was anyone else present besides the participants and researchers?  No; each interviewee was interviewed individually. Page 7 |
| 1. Description of sample | What are the important characteristics of the sample? E.g. demographic data, date  The characteristics of the interviewees are described, including their gender, age, academic background, professional title, years of experience and region in Table 1. Page 6-7 |
| **Data collection** | |
| 1. Interview guide | Were questions, prompts, guides provided by the authors? Was it pilot tested?  The main line of the interview guide revolved around the process of the registry study (Fig 1). The schedule of questions was formulated by the research team (FL, TYZ, YMZ, YYZ and ZXY) and includes 13 questions in 3 subjects (S2 Table). Page 7-8 |
| 1. Repeat interviews | Were repeat interviews carried out? If yes, how many?  All 14 participants completed two interviews. Page 8 |
| 1. Audio/visual recording | Did the research use audio or visual recording to collect the data?  The interview recordings were transcribed using the iFlytek Sr501 smart voice recorder. Page 9 |
| 1. Field notes | Were field notes made during and/or after the interview or focus group?  No. |
| 1. Duration | What was the duration of the interviews or focus group?  The interview sessions averaged approximately 32 minutes for each participant. Page 9 |
| 1. Data saturation | Was data saturation discussed?  The limitation part discussed the data saturation. Page 28 |
| 1. Transcripts returned | Were transcripts returned to participants for comment and/or correction?  Transcripts were submitted to the interviewees for confirmation. Page 9 |
| **Domain 3: Analysis and findings**  **Data analysis** | |
| 1. Number of data coders | How many data coders coded the data?  FL and TYZ coded the data independently. Any differences arising from the whole work were resolved by consensus. YQX and XFL participated in finalizing the code structure. Page 9 |
| 1. Description of the coding tree | Did authors provide a description of the coding tree?  Table 2 presents the thematic framework of the themes. Page 10-13 |
| 1. Derivation of themes | Were themes identified in advance or derived from the data?  Themes were identified in advance. |
| 1. Software | What software, if applicable, was used to manage the data?  No software was used. |
| 1. Participant checking | Did participants provide feedback on the findings?  Participant feedback was not undertaken. |
| **Reporting** | |
| 1. Quotations presented | Were participant quotations presented to illustrate the themes / findings? Was each quotation identified? E.g. participant number  Participant quotations were used to illustrate themes, with participants given numbers and title, to anonymize their identity. |
| 1. Data and findings consistent | Was there consistency between the data presented and the findings?  We believe there is consistency between the presented data and the findings. |
| 1. Clarity of major themes | Were major themes clearly presented in the findings?  Yes, see Overview in Results section. |
| 1. Clarity of minor themes | Is there a description of diverse cases or discussion of minor themes?  Within the major themes we have also discussed separate sub-themes. |
