# Supplementary material for: Practitioners’ perspectives on acupuncture treatment for postpartum depression: A qualitative study
Source: PLoS One. 2023 Mar 3;18(3):e0282661. doi: 10.1371/journal.pone.0282661 (PMC9983845; doi:10.1371/journal.pone.0282661)
Supplement: S2 Table — (DOCX) [file pone.0282661.s002.docx]

**Supporting table 2 Interview outline**

| **1. Subject one: Patient acceptance and compliance** | |
| --- | --- |
| 1.1 | How are PPD patients recruited? |
| 1.2 | How is the acceptance of acupuncture treatment in PPD patients? |
| 1.3 | What are the factors that influence patient acceptance? |
| 1.4 | How is compliance with acupuncture treatment in PPD patients? |
| 1.5 | What are the factors that influence patient compliance? |
| **2. Subject two: Acupuncture treatment for PPD** | |
| 2.1 | What is your acupuncture regimen for PPD? |
| 2.2 | What are the common complaints and symptoms of patients with PPD treated with acupuncture? |
| 2.3 | What is the overall effect of acupuncture on PPD? |
| 2.4 | What are the factors that affect the effectiveness of acupuncture? |
| 2.5 | How safe is acupuncture treatment? |
| **3. Subject three: Advantages and drawbacks of acupuncture treatment** | |
| 3.1 | What do you think are the advantages and drawbacks of acupuncture for PPD? |
| 3.2 | How do you think acupuncture can be optimized for PPD? |
| 3.3 | Could you share some successful or failed cases that made an impression on you? |
