# Supplementary material for: Practitioners’ perspectives on acupuncture treatment for postpartum depression: A qualitative study
Source: PLoS One. 2023 Mar 3;18(3):e0282661. doi: 10.1371/journal.pone.0282661 (PMC9983845; doi:10.1371/journal.pone.0282661)
Supplement: Attachment — Submitted filename: Response to Reviewers.docx [file pone.0282661.s003.docx]

Dear Editors and Reviewers,

We express our sincere thanks for the comments concerning our manuscript, entitled “Practitioners’ perspectives on acupuncture treatment for postpartum depression: A

qualitative study” (ID: PONE-D-22-18861). The comments are all valuable and very helpful for improving our paper. We have revised the manuscript and would like to resubmit it for your consideration. We have addressed the comments raised by the reviewers. Point-by-point responses to the reviewers’ comments are listed below this letter.

We hope that the revised version of the manuscript is now acceptable for publication in your journal.

I look forward to hearing from you soon.

With best wishes,

Yours sincerely,

Zhuoxin-Yang

**Editor’s Comments:**

I think the main problem of the manuscript is that the report on some key issues is not detailed, for example, the sample size estimation method, the sampling method and the professional background of the interviewees are not explained in detail. As the author mentioned in the discussion, if the sampling method is not random, or if the interviewee's experience of acupuncture treatment for PPD is not enough, the results of the interview may not be representative, so the conclusion needs to be reconsidered. At the same time, the author's report the results of data analysis is too complicated. It is suggested to give a table to summarize the theme, sub theme and codes extracted from the interview, and then explain and analyze them one by one. Although the author mentioned the report of the study was guided by the COREQ, the checklist should be attached as an appendix and the content of the manuscript should be checked again according to the checklist.

Please report the qualitative study according to the consolidated criteria for reporting qualitative health research checklist (COREQ), which you may find in EQUATOR website (https://www.equator-network.org/) or in below publication:

Tong A, Sainsbury P, Craig J. Consolidated criteria for reporting qualitative research (COREQ): a 32-item checklist for interviews and focus groups. Int J Qual Health Care. 2007; 19: 349–357; <https://doi.org/10.1093/intqhc/mzm042>

**Replies to the Editor**

Comment 1: I think the main problem of the manuscript is that the report on some key issues is not detailed, for example, the sample size estimation method, the sampling method and the professional background of the interviewees are not explained in detail. As the author mentioned in the discussion, if the sampling method is not random, or if the interviewee's experience of acupuncture treatment for PPD is not enough, the results of the interview may not be representative, so the conclusion needs to be reconsidered.

Response: Thank you for pointing out the sampling issue. As far as we are aware, the use of acupuncture to treat PPD in China is still fairly limited; hence, the number of experienced acupuncturists is small. This study is embedded within the multicentre registry trial, allowing us to contact acupuncturists with experience in the treatment of PPD. In the revised manuscript, we have added basic respondent inclusion criteria. We did our best to invite all competent frontline acupuncturists to participate, but information saturation cannot be guaranteed, as explained in the discussion section.

Comment 2: The author's report the results of data analysis is too complicated. It is suggested to give a table to summarize the theme, sub theme and codes extracted from the interview, and then explain and analyze them one by one.

Response: Thank you for your suggestion. We have supplemented the table (Table 2) in the revised manuscript. Page 10-13

Comment 3: Although the author mentioned the report of the study was guided by the COREQ, the checklist should be attached as an appendix and the content of the manuscript should be checked again according to the checklist.

Response: At your request, we checked the content of the manuscript again according to the COREQ checklist, and the revised manuscript has been supplemented with the checklist as an appendix.

**Reviewers' comments:**

Reviewer #1: This thesis is a subsidiary study of the Clinical Registry Study of Acupuncture for PDD. The study used a qualitative analysis to interview 14 clinicians within the registry system. The subject matter was interesting, but there were some methodological and writing issues.

1. All interviews for the study were conducted by independent authors. However, the process of interview outline formation was not described. Were other members of the team involved in relevant discussions? If so, the basic information about these members needs to be included.

2. How were the study participants identified? Were the 16 invited physicians all frontline therapists for this registered study? If not, what sampling principles were followed to select these 16 physicians?

3. The process of coding extraction and formation is very important, but is not described. This reduces the reliability of the study.

4. Figure 1 is unnecessary and it does not provide more information than the subheadings.

5. It is recommended to create an expanded flowchart. The flowchart could start with patient recruitment and go through treatment, data collection, and up to the completion of the clinical protocol and then out of the group. Include details of the questions for each process. This will be more informative.

6. The discussion is too superficial and does not demonstrate the authors' interest in the core issues of the study. Not all issues addressed in the paper need to be discussed equivalently. The author should have a notion of the importance of all issues, and relatively minor issues do not need to occupy equal space. This could have been a reason to reject the paper, but the work itself is valuable. It would be nice to read a more concise discussion.

7. This study is an affiliated study to a registered clinical study. What does the study recommend for the methodology of similar registry studies thereafter? It would be more informative.

**Replies to Reviewer #1**

Comment 1: All interviews for the study were conducted by independent authors. However, the process of interview outline formation was not described. Were other members of the team involved in relevant discussions? If so, the basic information about these members needs to be included.

Response: Thank you for your query. The revised manuscript supplements the interview outline formation process. The research team (FL, TYZ, YMZ, YYZ, and ZXY) participated in the formulation of the research questions, which are mentioned in the revised manuscript. Page7-8

Comment 2: How were the study participants identified? Were the 16 invited physicians all frontline therapists for this registered study? If not, what sampling principles were followed to select these 16 physicians?

Response: We apologize that we did not clarify the selection criteria for the respondents.

The following revisions were added to the manuscript: Based on a purposive sampling strategy, all frontline acupuncture practitioners in the registry study who met the following criteria were invited to participate in the qualitative interview study: practitioners who were involved in the clinical trial for more than 1 year and those who treated at least 20 patients. Ultimately, 16 acupuncturists from 7 Class-A tertiary hospitals met the requirements: One acupuncturist declined to participate, 1 did not respond, and 14 agreed to participate. Page 6

Comment 3: The process of coding extraction and formation is very important, but is not described. This reduces the reliability of the study.

Response: Thank you for your comment. The process of coding extraction and formation is summarized in a table (Table 2) that has been added to the revised manuscript. Page 10-13

Comment 4: Figure 1 is unnecessary and it does not provide more information than the subheadings.

Response: Thank you for your suggestion. We have removed Figure 1 in the revised manuscript.

Comment 5: It is recommended to create an expanded flowchart. The flowchart could start with patient recruitment and go through treatment, data collection, and up to the completion of the clinical protocol and then out of the group. Include details of the questions for each process. This will be more informative.

Response: Thank you very much for your constructive advice. Following your guidance, we have supplemented the flowchart as Figure 1 in the revised manuscript. Page 8

Comment 6: The discussion is too superficial and does not demonstrate the authors' interest in the core issues of the study. Not all issues addressed in the paper need to be discussed equivalently. The author should have a notion of the importance of all issues, and relatively minor issues do not need to occupy equal space. This could have been a reason to reject the paper, but the work itself is valuable. It would be nice to read a more concise discussion.

Response: We appreciate the critical comment. Based on the core issue of the clinical feasibility of acupuncture for PPD, we have reworked the Discussion section to focus on the advantages and drawbacks of acupuncture for PPD as well as suggestions for future improvements. We hope that the updated Discussion section is more concise and concentrated.

Comment 7: This study is an affiliated study to a registered clinical study. What does the study recommend for the methodology of similar registry studies thereafter? It would be more informative.

Response: We apologize that this study did not involve the issue of registry research methodology, and we thank you for your suggestion to help us improve the design of future research protocols.

**Reviewers' comments:**

Reviewer #2: In the statements from the acupuncture practitioners, there are some minor English errors.

Page 18 comment on top of the page ..."They prefer to adopt it because we are confident in our skills." I would suggest adding [acupuncture] after the work "it" so the meaning is clear.

page 20: "Time is the most important factor, as patients need to breastfeed. In fact, there were not a few patients who came with their children, which must be a great obstacle" In this statement, it is unclear whether the breastfeeding or having children at the appointment is the issue. While this is a quote, some edits are needed to clarify what the issue is.

page 26: "Time is the most important factor, as patients need to breastfeed. In fact, there were not a few patients who came with their children, which must be a great obstacle." For this sentence, I believe the "he" needs to be replaced with "she"

Page 27: "Because of the wide variety of acupuncture treatments, multiple acupuncture therapies can be combined to accomplish the goal of synergistic and synergistic enhancement." There must be a typo here. Synergistic does not need to be used twice.

**Replies to Reviewer #2**

Thank you very much for pointing out the errors in English usage. The above errors have been corrected in the revised manuscript. In addition, this manuscript has been edited and proofread by AJE for language-related concerns.

**Reviewers' comments:**

Reviewer #3: It is interesting that authors conducted a qualitative study of practitioners’ perspectives on acupuncture treatment for postpartum depression, I think several concerns listed below will need to be addressed to improve paper quality.

1. The sample size ( n = 14) was too small to generalize the findings of this study. Please provide a rationale for generalizing your findings

2. Please provide the evidence that participants are specialist in acupuncture treatments for postpartum depression.

3. Please provide the raw data summarizing participant’s answers to each question as a supporting file.

4. Please provide the ethical approval date.

**Replies to Reviewer #3**

Comment 1: The sample size ( n = 14) was too small to generalize the findings of this study. Please provide a rationale for generalizing your findings.

Response: Thank you for pointing out the sample size issue. As far as we are aware, the use of acupuncture to treat PPD in China is still fairly limited; hence, the number of experienced acupuncturists is small. This study is embedded within the multicentre registry trial, allowing us to contact acupuncturists with experience in the treatment of PPD. In the revised manuscript, we have added basic respondent inclusion criteria. We did our best to invite all competent frontline acupuncturists to participate, but information saturation cannot be guaranteed, as explained in the discussion section.

Nevertheless, the findings drawn through the rigorous data analysis process are still helpful in answering the research questions.

Comment 2: Please provide the evidence that participants are specialist in acupuncture treatments for postpartum depression.

Response: Thank you for your comment. All the participants were from the multicentre registry study. According to the research plan, an acupuncture practitioner needed a physician's qualification certificate and licence as well as at least three years of work experience in the acupuncture department. Before the study, the practitioner received training on PPD acupuncture operation standards. In addition, we have supplemented the inclusion criteria in the revised manuscript as follows: practitioners who were involved in the clinical trial for more than 1 year and those who treated at least 20 patients. Page 6

Comment 3: Please provide the raw data summarizing participant’s answers to each question as a supporting file.

Response: Thank you for your comment. In ethically reviewed research protocols, the informed consent forms contain undisclosed respondents' raw data. Data cannot be shared publicly because recordings of the conversations are private and contain potentially identifying and personal information. Data are available from the Ethics Committee of Shenzhen Traditional Chinese Medicine Hospital for researchers who meet the criteria for access to confidential data. The data underlying the results presented in the study are available via the following email address: szszyyll@126.com.

Comment 4: Please provide the ethical approval date.

Response: Thank you for your careful reading. We have added the ethical approval date to the revised manuscript. Page 7
