# Supplementary material for: Practitioners’ perspectives on acupuncture treatment for postpartum depression: A qualitative study
Source: PLoS One. 2023 Mar 3;18(3):e0282661. doi: 10.1371/journal.pone.0282661 (PMC9983845; doi:10.1371/journal.pone.0282661)
Supplement: Attachment — Submitted filename: Response to Reviewers20221214.docx [file pone.0282661.s004.docx]

Dear editors and reviewers,

We express our sincere thanks for the comments concerning our manuscript, entitled “Practitioners’ perspectives on acupuncture treatment for postpartum depression: A

qualitative study” (ID: PONE-D-22-18861). The comments are all valuable and very helpful for improving our paper. We have revised the manuscript and would like to resubmit it for your consideration. We have addressed the comments raised by the reviewers. Point-by-point responses to the reviewers’ comments are listed below this letter.

We hope that the revised version of the manuscript is now acceptable for publication in your journal.

I look forward to hearing from you soon.

With best wishes,

Yours sincerely,

Zhuoxin-Yang

**Reviewers' comments**

Reviewer #3: Thank you for your response to my comments. However, I am not satisfied with your response. The quality of this manuscript is low due to small sample size and the ambiguity of the research process. You should include frontline acupuncture practitioners who were not involved in the registry study.

**Replies to Reviewer #3**

We apologize that our response did not meet your expectations. Please allow us to elaborate further on the sample size. Power calculations determine the sample size (N) necessary to demonstrate effects of a certain magnitude from an intervention in quantitative studies. However, for qualitative interview studies, no similar standards for the assessment of sample size exist. Compared to quantitative research, qualitative research typically employs a small sample size. However, the small sample size does not indicate low quality of the study. Qualitative research is largely exploratory and aims to deepen our understanding of a topic or phenomenon by providing new insights. Research with social constructivist roots, where knowledge is considered partial, intermediate, and dependent on the situated view of the researcher, does not support the idea that qualitative studies ideally should comprise a “total” amount of facts. Some scholars propose the concept of “information power”^[1]^, which suggests that the more information the sample holds that is relevant for the actual study, the lower the number of participants that is needed. In this regard, sample adequacy, data quality, and the variability of relevant events are often more important than the number of participants.

Regarding your suggestion to include frontline acupuncture practitioners who were not involved in the registry study, this approach was inconsistent with the goal of our research. Only acupuncturists with sufficient experience in the treatment of postpartum depression could adequately comment on this subject. Based on the results of the preliminary exploratory research, we will invite more frontline acupuncturists if we conduct consensus research in the future.

Regarding your concern about the ambiguity of the research procedure, we must admit that due to the subjectivity and flexibility of qualitative research, quality control and evaluation are more difficult than they are in quantitative research. We have attempted to adhere to the items of the Consolidated Criteria for Reporting Qualitative Research (COREQ) to ensure that reflexivity, transparency and interactivity were implemented in the design, conduct and reporting stages of the qualitative interviews.

[1] Malterud K, Siersma V D, Guassora A D. Sample size in qualitative interview studies: guided by information power[J]. Qualitative health research, 2016, 26(13): 1753-1760.

**Reviewers' comments**

Reviewer #4: GENERAL: The text is not paged for ease of assessment.

METHODS: Under study design, the authors should define what they mean by class A tertiary hospitals. What do the authors mean by postdoc? Under ethical considerations, how were written informed consents obtained from the participants that resided for away and had to be interviewed by telephone?

REFERENCES: This is ok.

**Replies to Reviewer #4**

Comment 1: Under study design, the authors should define what they mean by class A tertiary hospitals.

Response: Thank you for your comment. Class A tertiary hospitals are the highest level of hospitals in mainland China and are classified in accordance with the Measures for Graded Management of Hospitals.

The classification of Chinese hospitals is a 3-tier system including primary, secondary and tertiary hospitals. A tertiary hospital is a comprehensive, referral, general hospital at the city, provincial or national level with a bed capacity exceeding 500. These hospitals are responsible for providing specialist health services, perform a larger role in medical education and scientific research and serve as medical hubs that provide care to multiple regions. A tertiary hospital is similar to a tertiary referral hospital in the West. Based on the level of service provision, size, medical technology, medical equipment, and management and medical quality, these 3 grades are further subdivided into 3 subsidiary levels: A, B and C.

Comment 2: What do the authors mean by postdoc?

Response: Thank you for your query. According to the consolidated criteria for reporting qualitative studies (COREQ) 32-item checklist, researchers should describe personal characteristics such as their credentials, occupation, gender, experience and training to show their reflexivity. This interview study was conducted by the first author, who was a postdoctoral fellow working in Shenzhen Traditional Chinese Medicine Hospital at the time of the study.

Comment 3: Under ethical considerations, how were written informed consents obtained from the participants that resided for away and had to be interviewed by telephone?

Response: Thank you for your query. Written informed consent was collected by post or fax from participants who were interviewed via telephone.

**Reviewers' comments**

Reviewer #5: The manuscript is concerned about the small sample size and results. In addition, the results are not quantified, making objective understanding difficult.

Acupuncture is indicated in the title, but the effect of acupuncture itself is not shown. Because of this, the correct word to include in the title would be “acupoint stimulation”.

In addition, It should be noted why were the acupuncture points used? For example, Historically, what are the benefits of each targeted acupuncture points for this symptoms?

Various techniques are used to stimulate the acupuncture points (e.g. auricular acupuncture, cupping, moxibustion) in the method paragraph. But it will make the outcome more difficult.

Please provide evidence that the title is true to show the effectiveness of acupuncture in the presence of other stimuli.

Write the method of stimulation details such as needle type, stimulation frequency or intensity, insert depth, and duration of acupuncture care. In addition, consider the differences in the stimuli from previous studies.

**Replies to Reviewer #5**

Thank you for your valuable comments. As you mentioned, the small sample size and the lack of quantitative results are disadvantages of qualitative research compared to quantitative research. Qualitative research aims to address questions concerned with developing an understanding of the meaning and experience dimensions of humans’ lives and social worlds. The data are analysed by summarizing, categorizing and interpreting, so the results are mainly expressed in words. Interpretation of the results should be considered within the context of qualitative research and the exploratory nature of the study.

Comment 1: Acupuncture is indicated in the title, but the effect of acupuncture itself is not shown. Because of this, the correct word to include in the title would be “acupoint stimulation”. Various techniques are used to stimulate the acupuncture points (e.g. auricular acupuncture, cupping, moxibustion) in the method paragraph. But it will make the outcome more difficult. Please provide evidence that the title is true to show the effectiveness of acupuncture in the presence of other stimuli.

Response: Thank you for your comments. The qualitative research is an adjunct to the clinical registry study of acupuncture for PPD, the objective of which is to assess the efficacy and safety of acupuncture in the treatment of mild to moderate PPD as well as the advantages of various types of acupuncture, including filiform needle acupuncture, electroacupuncture, and auricular acupuncture. Since this was a real-world study, physicians could select different acupuncture treatment plans based on the conditions of individual patients. Additionally, combination therapies, such as cupping and moxibustion, could be used. Patients undergoing different treatment plans naturally created distinct cohorts, allowing for the improvement of treatment plans through statistical analysis.

In this study, most of the patients only received filiform needle acupuncture. With regard to the effect of acupuncture, our team published a Chinese paper^[2]^ based on a mid-term analysis.

[2]Yan B, Yang Z X, Cui L L, et al. Mild and moderate postpartum depression treated with acupuncture of Tiaoren Tongdu: a real world study[J]. Zhongguo Zhen jiu= Chinese Acupuncture & Moxibustion, 2021, 41(8): 877-882.

Comment 2: In addition, It should be noted why were the acupuncture points used? For example, Historically, what are the benefits of each targeted acupuncture points for this symptoms?

Response: Thank you for your query. Tiaoren Tongdu refers to a collection of acupoints selected primarily from the Ren and Du meridians. It was developed by our research team based on classical literature and clinical experience to treat neuropsychiatric disorders. Its efficacy in treating poststroke depression and insomnia has been demonstrated through clinical research.

According to the current literature review on the treatment of depression by acupuncture^[3]^, the core points (GV20, GV29, PC6, HT7, LI4, ST36, SP6, LR3) for the treatment of depression are all included in the Tiaoren Tongdu. Therefore, Tiaoren Tongdu represents the conventional acupuncture therapy strategy for depression.

GV20 and GV29 are located in the Du meridian (Governor Vessel meridian) on the head and have the effect of regulating the mind (Shen) and relieving depression. CV12, CV6 and CV4 are located in the Ren meridian (Conception Vessel meridian), adjacent to the uterus, and can replenish vitality and kidney essence.The Du meridian is dominant to the Yang meridian, while the Ren meridian is dominant to the Yin meridian. Ren and Du meridian acupuncture can balance the Yin and Yang of the human body.

According to traditional Chinese medicine, the heart controls mental activities. PC6 lies on the pericardium meridian, whereas HT7 is on the heart meridian. These acupoints are frequently used to nourish the mind and calm the nerves.

Traditional Chinese medicine suggests that depression is mostly related to the stagnation of liver qi. LR3 and LI 4 are typical combinations of acupoints that can help regulate liver qi. Traditional Chinese medicine also suggests that the spleen and stomach are the foundation of acquired life and the source of qi and blood. SP6 is located in the spleen meridian, which has the ability to generate blood. ST36 is located in the stomach meridian, which helps replenish qi. The combination of two acupoints can replenish qi and blood. Therefore, this combination is suitable for the constitution of postpartum women.

[3]Sun Z Y, Shan X J, Huang X Y, et al. Visual analysis of literature knowledge structure and acupoint matching rules of acupuncture for depression[J]. Zhongguo Zhen jiu= Chinese Acupuncture & Moxibustion, 2021, 41(9): 1049-1054.

Comment 3: Write the method of stimulation details such as needle type, stimulation frequency or intensity, insert depth, and duration of acupuncture care. In addition, consider the differences in the stimuli from previous studies.

Response: Thank you for your comment. The needle type we used was 0.3 mm × 40 mm/0.3 mm × 75 mm. For the GV20 and GV29 acupoints, the needles are inserted horizontally at 0.5-0.8 cun to induce a sensation of soreness (de qi). For the PC6, HT7, LI4, ST36, SP6, and LR3 acupoints, the needles are inserted vertically to a depth of 0.8-1.0 cun. For the CV4, CV6 and CV12 acupoints, the needles are inserted obliquely at 1.0-1.2 cun. Acupuncturists rotate and lift the needles for 30 s to achieve de qi. The treatment period is 8 weeks, and the treatment frequency is three times per week, for a total of 24 acupuncture treatments over 8 weeks.

Conventional filiform needle acupuncture therapy was used in this study, which does not differ from the stimuli in previous studies.
