# Supplementary material for: Practitioners’ perspectives on acupuncture treatment for postpartum depression: A qualitative study
Source: PLoS One. 2023 Mar 3;18(3):e0282661. doi: 10.1371/journal.pone.0282661 (PMC9983845; doi:10.1371/journal.pone.0282661)
Supplement: Attachment — Submitted filename: Response to Reviewers20230208.docx [file pone.0282661.s005.docx]

Dear editors and reviewers,

We express our sincere thanks for the comments concerning our manuscript, entitled “Practitioners’ perspectives on acupuncture treatment for postpartum depression: A

qualitative study” (ID: PONE-D-22-18861). The comments are all valuable and very helpful for improving our paper. We have revised the manuscript and would like to resubmit it for your consideration. We have addressed the comments raised by the reviewers. Point-by-point responses to the reviewers’ comments are listed below this letter.

We hope that the revised version of the manuscript is now acceptable for publication in your journal.

I look forward to hearing from you soon.

With best wishes,

Yours sincerely,

Zhuoxin-Yang

**Journal Requirements:**

Please review your reference list to ensure that it is complete and correct. If you have cited papers that have been retracted, please include the rationale for doing so in the manuscript text, or remove these references and replace them with relevant current references. Any changes to the reference list should be mentioned in the rebuttal letter that accompanies your revised manuscript. If you need to cite a retracted article, indicate the article’s retracted status in the References list and also include a citation and full reference for the retraction notice.

**Additional Editor Comments:**

The sample size of qualitative research generally adopts the principle of information saturation. The author also fully mentions the uncertainty of the results caused by the sampling method in the limitations section. I suggest the authors to add whether (all or most of) the respondents are from the same hospital. If so, please also explain the general information of acupuncture treatment for postpartum depression in that hospital.

**Replies to Editor:**

Thank you for your suggestion. In the method section (Participants and setting /p5), we mentioned that the respondents were from 7 hospitals, and in the results section (Acupuncture treatment methods/p18), we introduced the general information of acupuncture treatment for PPD.

The references have been reviewed to ensure accuracy.
